# Applications of three-dimensional carbon nanotube networks

**DOI:** 10.3762/bjnano.6.82

**Published:** 2015-03-23

**Authors:** Manuela Scarselli, Paola Castrucci, Francesco De Nicola, Ilaria Cacciotti, Francesca Nanni, Emanuela Gatto, Mariano Venanzi, Maurizio De Crescenzi

**Affiliations:** 1 Dipartimento di Fisica, Università di Roma Tor Vergata, Via della Ricerca Scientifica 1, 00133 Roma, Italy; 2 Università di Roma Niccolò Cusano (INSTM-UdR), Via Don Carlo Gnocchi 3, 00166 Roma, Italy; 3 Dipartimento di Ingegneria dell'Impresa, Università di Roma Tor Vergata (INSTM-UdR Roma Tor Vergata), Via del Politecnico 1, 00133 Roma, Italy; 4 Dipartimento di Scienze e Tecnologie Chimiche, Università di Roma Tor Vergata, Via della Ricerca Scientifica 1, 00133 Roma, Italy

**Keywords:** carbon nanotube sponge, electrochemical, hydrophobicity, lipophilicity

## Abstract

In this paper, we show that it is possible to synthesize carbon-based three-dimensional networks by adding sulfur, as growth enhancer, during the synthesis process. The obtained material is self-supporting and consists of curved and interconnected carbon nanotubes and to lesser extent of carbon fibers. Studies on the microstructure indicate that the assembly presents a marked variability in the tube external diameter and in the inner structure. We study the relationship between the observed microscopic properties and some potential applications. In particular, we show that the porous nature of the network is directly responsible for the hydrophobic and the lipophilic behavior. Moreover, we used a cut piece of the produced carbon material as working electrode in a standard electrochemical cell and, thus, demonstrating the capability of the system to respond to incident light in the visible and near-ultraviolet region and to generate a photocurrent.

## Introduction

In the last years, there has been growing interest in developing natural and synthetic three-dimensional architectures rather than two-dimensional ones because of the increase of active surface area throughout the entire 3D structure. Hydrogels, organogels, and aerogels based on silica [1] or carbon [2] and consisting of micro-, and macroscopic assemblies are reported in the literature. In particular, self-sustaining assemblies that show high porosity [3], structural stability, and good electrical conductivity [4] are the best candidates for environmental applications such as filtration [5], separation [6], biological sensors [7], and oil-spill remediation [8] but also as mechanical actuators [9], catalytic supports [10], and super capacitors [11]. In this research field, architectures based on carbon nanotubes (CNTs) are the focus of intense research activity [2]. Since CNTs are one-dimensional structures with well-known electrical and mechanical properties, they are the ideal building blocks for constructing three-dimensional random meshes from their overlapping. Recently, Gui and co-workers [6] fabricated CNT-sponges through a chemical vapor deposition (CVD) process during which a catalyst precursor (ferrocene) dissolved in dichlorobenzene, which acted as carbon precursor, was injected in the reactor chamber. They obtained millimeter-thick CNT-assemblies in which individual nanotubes were stacked in a random manner to form the bulk material.

Similarly, in this paper, we show that through carrying out a CVD synthesis with different precursors it is possible to synthesize three-dimensional carbon networks consisting of randomly interconnected nanostructures. The bulk carbon nanostructures display a structural flexibility rarely observed in other high-porosity materials, e.g., bulk carbon aerogels [12] or aligned CNT arrays [3]. The pristine CNT-sponges are super-hydrophobic (i.e., a water contact angle greater than 150°) and oleophilic as reported recently [13–14]. Therefore, in this paper, we show that the structural and electronic features of the network are directly responsible for the hydrophobic and the lipophilic behavior.

In addition, to test the capability of the system to respond to incident light and generate a photocurrent, we cut a piece of the CNT-sponge, which has a self-sustainable structure, and used it as the working electrode in a standard electrochemical cell. In this manner, it was possible to register a good photo-response of the CNT-network in the visible and near-ultraviolet range.

## Results and Discussion

**Figure 1:**
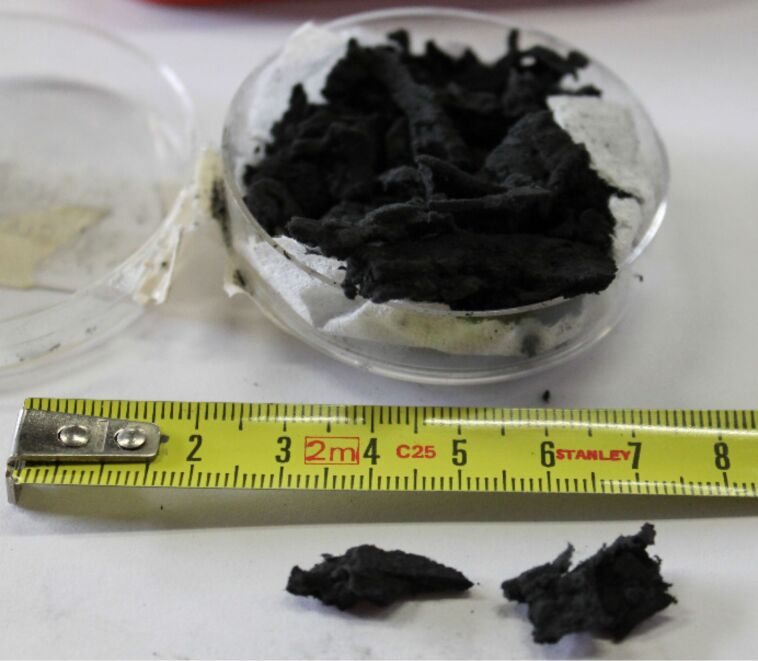
Photograph of a dish containing CNT-sponges, and two cut pieces of few cubic mm.

The produced CNT-sponges are light and porous, and can be cut into pieces of the desired size as shown in Figure 1. The micro-porosity of the synthesized material is responsible for its very low density of about 15 mg/cm^3^ and its good conductivity (electrical resistance of about 40 Ω·cm^−1^). These values are comparable to those reported in the literature for similar samples [6].

**Figure 2:**
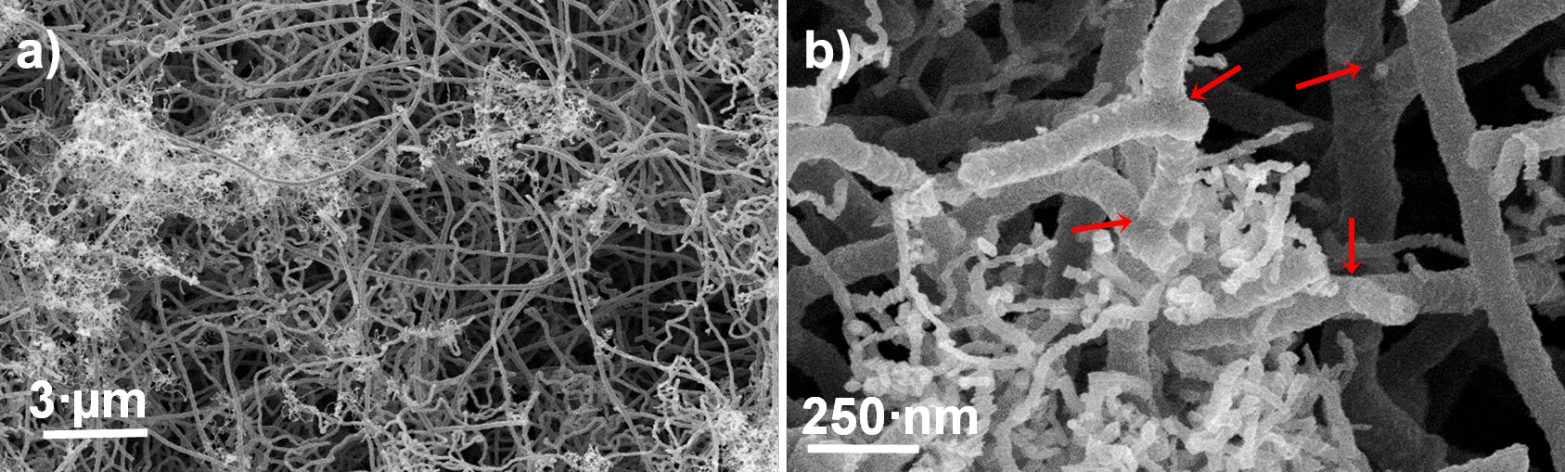
SEM micrographs showing the entangled structure of the network acquired at two different magnifications (a,b). The red arrows in panel (b) point to junctions between CNTs.

Scanning electron microscopy (SEM) analysis of the interior of the samples reveals that the material is made of randomly self-assembled, long and interconnected tubular nanostructures, with pore sizes from several nanometers to a few micrometers (Figure 2a). The high number of interconnections indicated by the arrows in Figure 2b is caused by the presence of topological defects in the carbon sp^2^ lattice that originate during the growth process. In particular, the addition of sulfur to the synthesis process induces the formation of pentagonal and heptagonal carbon rings [15–16] that favors the bent geometry of the nanostructures. No structural differences are observed along the whole sample within this morphological characterization.

**Figure 3:**
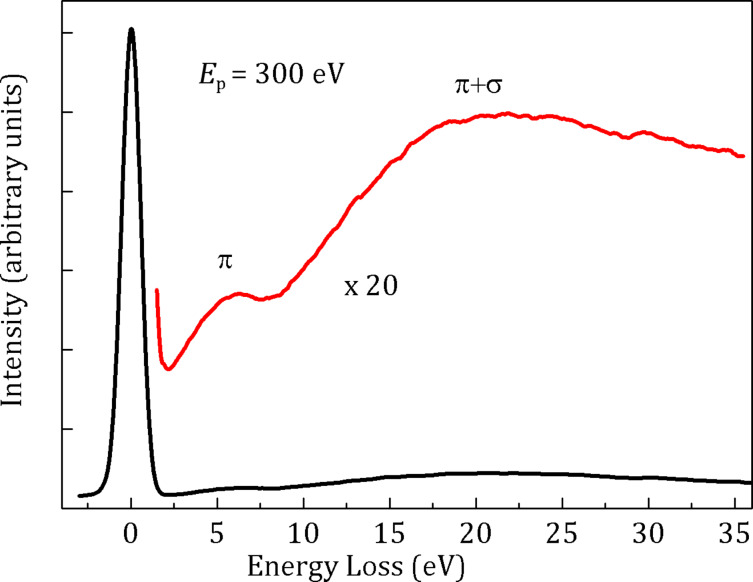
Electron energy loss spectra (*E*_p_ = 300 eV) obtained on the CNT-sponge. The π and π + σ plasmons have been emphasized for better view (red line).

In the case of the sample reported in Figure 2, the statistical analysis gives a double distribution of the external diameter centered at 91 ± 3 nm, and 418 ± 5 nm, respectively. The length of the tubes can vary from few nanometers to millimeters, and the CNTs have a high number of walls as evidenced by a transmission electron microscopy studies described in [13]. SEM micrographs obtained at higher magnification evidence the presence of interconnected and curled CNTs as shown in Figure 2b. The electron energy loss spectroscopy analysis performed in reflection mode further supports the predominance of C-sp^2^ hybridization in the nanotubes, as indicated by the electron microscopy studies. In fact, the spectrum in Figure 3, displays two peaks at 6 eV and 24.5 eV. These are contributions coming from the π and π + σ plasmons, respectively, of the sp^2^ lattice [17]. In particular, the energy positions of both features are downshifted in energy loss compared to those of the highly oriented pyrolytic graphite (7 eV and 28 eV, respectively). This behavior is due to the low dimensionality of the system similar to that reported for multi-walled CNTs [18].

**Figure 4:**
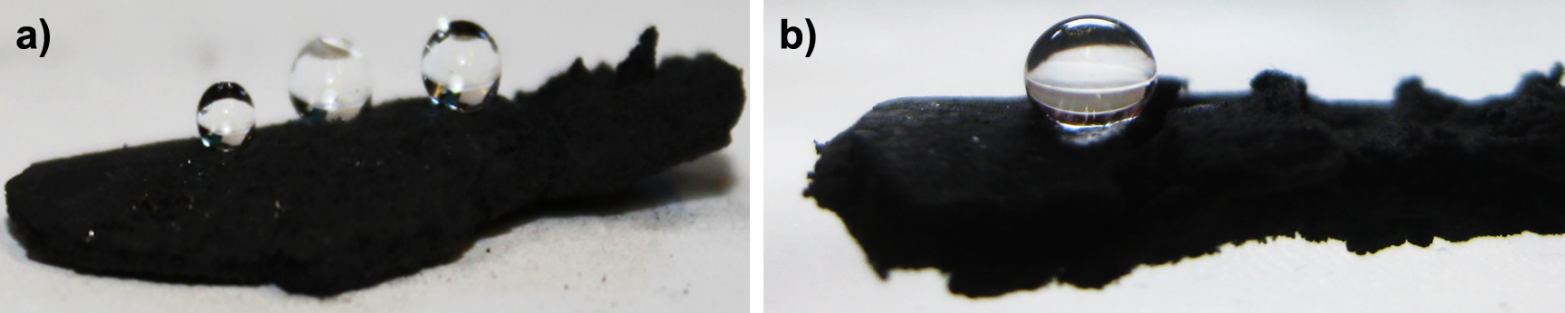
Photographs of water droplets of different volumes (a) and contact angle profile of a single drop (b) on the bulk material.

The micro-porosity of the synthesized material is responsible of its low weight and its capability to sustain high compression loads as recently reported for our samples [14]. Furthermore, two interesting properties that originate from the high porosity and the presence of numerous interconnected one-dimensional nanostructures are the hydrophobicity and oleophilicity. To better characterize the hydrophobicity one can measure the advanced static contact angle at room temperature for water droplets of different volumes ranging from 5 to 20 µL, as shown in Figure 4a.

**Figure 5:**
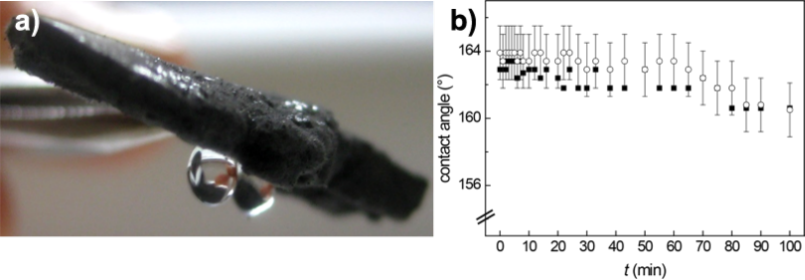
Stability of the super-hydrophobic state. No roll-off angle was measured, even when the substrate is turned upside down (a). Variation of the measured contact angle for a 10 µL water droplet as a function of the time, the vertical scale has been expanded for better view (b).

The presence of a composite solid–liquid–air interface explains the high value of the measured contact angle (Θ = 175°), as evaluated in Figure 4b, with no observable roll-off angle, even when the substrate is turned upside down, see Figure 5a. Therefore, we infer that the contact angle hysteresis is sufficiently high to pin the water droplet on the MWCNT surface. It is possible to estimate the adhesive force in length units of a surface in contact with water from the equation [19]:

[1]
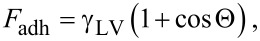


where γ_LV_ denotes the surface tension of the liquid–vapor (LV) interface for water γ_LV_ = 72.5 mN/m, and Θ is the measured contact angle (Θ = 175°). The estimated adhesion force of the water droplet (20 µL) reported in Figure 4b, is about 50 µN.

Measuring the variation of the static contact angle as a function of the time at room temperature shows a reduction of the initial value of less than 2% after 100 min, due to water evaporation. This proves the stability of the super-hydrophobic state (Figure 5b). These findings indicate that the CNT-sponge wettability is well described by a Cassie–Baxter model [20] for which a quite rough surface allows air trapping and ensures the high contact angle measured. In particular, in such a system pores in the random network (i.e., void fraction) favor air trapping due to the strong capillary force that the surface exerts on the liquid. The water drop can be viewed as sitting on a composite surface consisting of solid and air. Therefore, one can describe the wetting properties of the sponge surface in the super-hydrophobic regime using the Cassie-Baxter equation [20]:

[2]
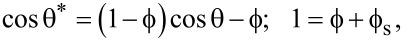


where 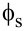 and 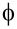 are the fractions of solid and air contacting the water droplet, θ* is the apparent contact angle, and θ is the Young’s contact angle of the surface, with cosθ defined as:

[3]
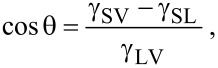


where γ_SV_, γ_SL_, and γ_LV_ denote the surface tension of the solid–vapor (SV), the solid–liquid (SL), and the liquid–vapor (LV) interface, respectively. If we insert in Equation 2 the Young’s contact angle measured experimentally for a MWCNT [21–22] (from Nanocyl, NC7000, diameter: 5–50 nm) random network film, 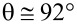, and as apparent contact angle the average value θ* = 175°, we obtain a surface air fraction of 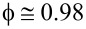.

This value indicates that the wet fractional area of the drop base in contact with the sponge surface is very low and thus large amount of air pockets formed, because of the fakir effect [23] induced by the high roughness of the CNT random network.

**Figure 6:**
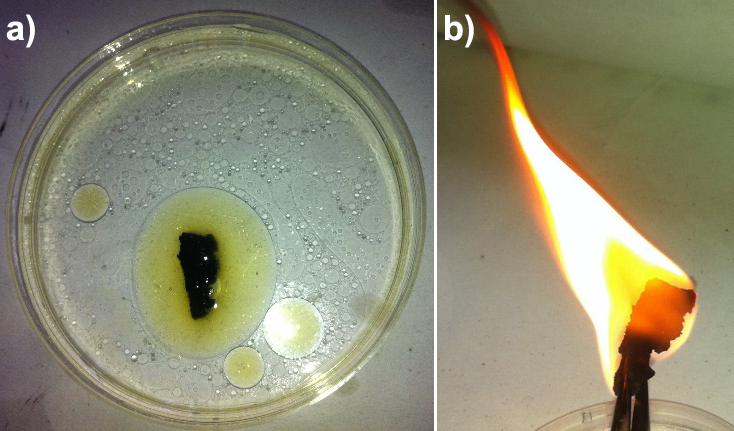
Burning and reuse of the CNT-sponge. Photograph of the starting of the oil-adsorption process (a), and burning after the sponge got saturated with oil (b).

On the other hand, the CNT-sponge shows a high absorption capacity towards oils (e.g., vegetable and mineral oil). The contact angle is significantly less than 90° and therefore the CNT-sponge is lipophilic [6]. In Figure 6a the beginning of the removal of mineral oil (from AGIP company, ISO46) spreading on the water surface using the as-prepared three-dimensional material is shown. It is possible to evaluate the adsorption capacity of the sponge, from the ratio between the final and initial weight after full adsorption [6]. As an example, a CNT-sponge of 2 mg is able to selectively uptake vegetable oil up to 16 times of its initial weight. The observed high value of the uptake efficiency can be ascribed to the presence of both (i) carbon sp^2^ species (e.g., nanofibers), characterized by a rough surface, and (ii) the high porosity. In fact, it is known that irregular surfaces make adsorption of organics much easier than smooth ones [24]. The adsorbed oil can easily be removed after the sponge is saturated simply through squeezing it from the sponge or through burning it. In Figure 6b, the oil-saturated sponge is ignited, the fire burns the oil and the sponge is ready to be reused.

**Figure 7:**
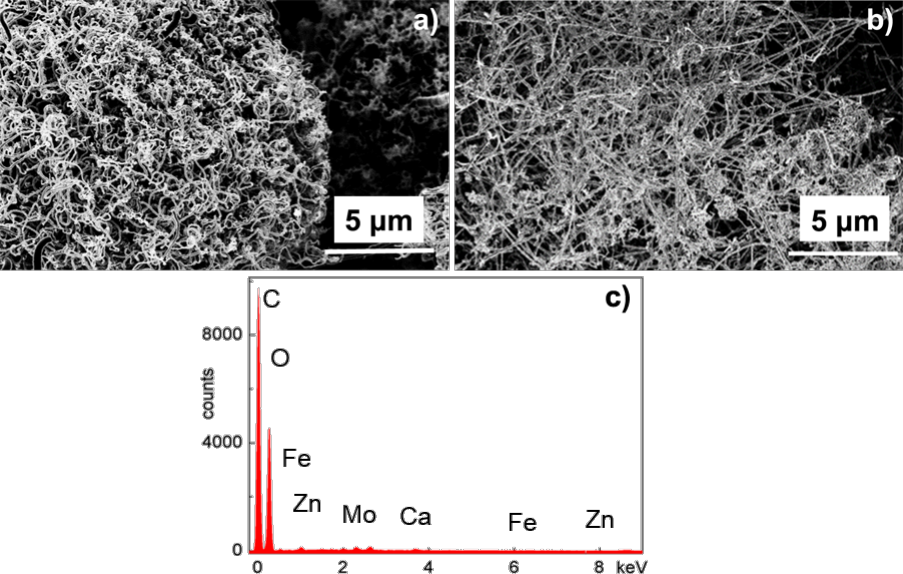
SEM micrographs of the CNT-sponge surface after one (a) and two (b) burning processes. Correspondent EDX spectrum collected from sample (a) showing some constituents of the adsorbed oil together with the main components of the CNT-sponge (c).

We investigated with SEM on the nanostructure properties before and after oil adsorption and the subsequent burning process. Figure 7a and Figure 7b show two SEM micrographs obtained from the surface of the CNT-sponge after one and two adsorption–burning processes, respectively. While the porous assembly appears almost entirely preserved, the nanostructures composing the network are partially covered by some oil residues. This finding is confirmed by EDX spectrum collected on the network of Figure 7a, that shows contributions from: the CNT-nanostructures (C and Fe), some of the constituents the oil used in the experiment (Zn, Mo) and small traces of Ca from water, Figure 7c. No sulfur signal was detected in the network, indicating that the sulfur content is too small. Therefore, we can assume that almost no sulfur was trapped in the network, in accordance to what was reported for similar CNT-networks [25].

A sponge-like multiwall-CNT framework was recently used as the catalytic layer of a counter electrode for a dye-sensitized solar cell (DSC) [26]. The CNT-sponge was directly transferred onto a conductive substrate to make a counter electrode (CE), exploiting its good electrical and mechanical properties. A photoelectric conversion efficiency of about 6.2% was achieved for the DSC with a CNT-sponge CE, compared to 7.6% of that with Pt CEs.

**Figure 8:**
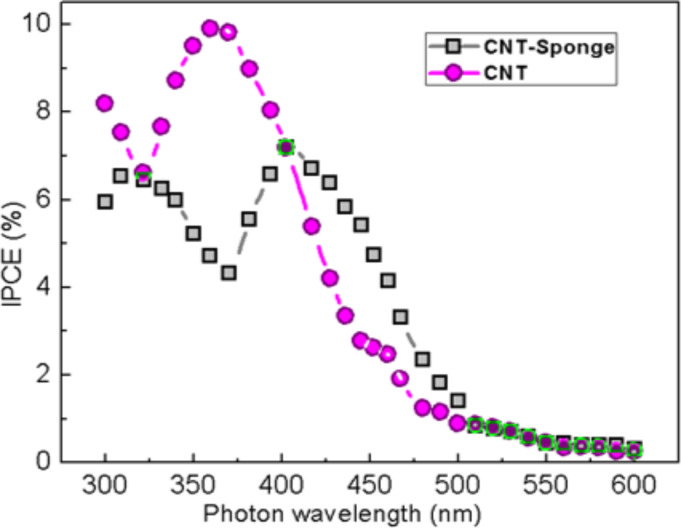
Incident-photon-to-current efficiency (IPCE, %) obtained from a MWCNT 2D film (purple circles), and for a CNT-sponge sample (grey squares) as function of incident photon wavelength.

Recently, our research group showed that 2D films made of pristine MWCNTs can be used as optically active medium for light energy conversion in a solar cell device [27]. Accordingly, we performed a similar measurement using a piece of CNT-sponge, which possesses a self-supporting structure, as the working electrode in a standard electrochemical cell. The photo-response was measured as a function of the incident photon wavelength and expressed in terms of IPCE (incident-photon-to-current efficiency) (Figure 8). In the same figure, the response obtained from a MWCNT film grown on a silicon substrate [27–29] is reported for comparison. The samples show a photo response to the incident light with a similar IPCE trend. It is noteworthy that the signal coming from the CNT-sponge has a maximum around 420 nm, red-shifted with respect to that obtained from CNTs film of around 360 nm. The observed difference can be ascribed to the highly complex microscopic structure of the sponge compared to that of the MWCNTs composing the film. Indeed, not only the CNT-sponge presents a marked variability in the tube diameter but also carbon few fibers are present in the network. These structural fluctuations give different response to incident light.

## Conclusion

A chemical vapor deposition process can be used to synthesize three-dimensional porous structures composed of CNTs. The obtained CNT material exhibits a sponge-like structure and has a low density. The capability to adsorb and remove different types of oil has been demonstrated and can be considered very interesting for environmental applications. In addition, the CNT network shows a good photo response to incident light in the visible and near ultraviolet range, thus proving its potential application in photon-energy conversion devices. The collected results enable us to predict that the produced CNT-sponges are an interesting example of nanostructured materials that may be employed in new emerging fields of applications such as environmental sector, sensing and electromechanical transduction. At the same time, a thorough study on the correlation between the observed properties and the structural and electronic features of the network is ongoing.

## Experimental

**Chemical vapor deposition process for the growth of 3D CNT networks:** The chemical vapor deposition process was carried out in a horizontal hot-wall quartz furnace. Prior to the experiment, argon gas was flushed into the growth chamber, to restore an ambient inert pressure (760 Torr). Ferrocene (2.3 wt %) and tiophene (1.5 wt %) were dissolved in ethanol to be used as catalyst and sulfur precursors, respectively. The obtained solution was placed in a 10 mL glass syringe and injected into the growth chamber at a constant rate of 7 mL/h through a flux of argon and acetylene (500/200 sccm), which act as gas carrier and carbon precursor, respectively. The vaporized solution and the gas mixture were injected through a stainless tube directly into the high temperature region of the quartz tube furnace. The CNT synthesis was carried out at a temperature of 900 °C, measured by an optical pyrometer.

**Microstructure investigation:** The morphology of the CNT-sponges was examined with a field emission gun scanning electron microscope (FEG-SEM, Leo Supra 35) equipped with energy dispersion spectroscopy (EDX).

**Electron energy loss analysis**: Electron energy loss (EELS) was recorded in reflection mode ex situ in an ultrahigh vacuum system (base pressure about 2 × 10^−10^ Torr) equipped with an electron gun (*E*_p_ = 300 eV, Δ*E* = 1.0 eV).

**Contact angle measurements:** Static advanced contact angles were measured from optical images and analyzed with the open-source software ImageJ.

**Evaluation of the oil-adsorption capacity:** A cut piece of CNT-sponge was laid down onto the surface of a vessel containing water over which drops of mineral oil (from AGIP Company, Italy) have been deposited. By measuring the ratio between the mass of the dry CNT bulk material and the mass after oil adsorption, the oil-adsorption capacity was evaluated. The adsorbed oil in the material was then removed burning it in air and then was used to repeat the above process for many cycles.

**Photocurrent measurements:** Photo-electrochemical measurements were carried out at room temperature with a standard three-arm photo-electrochemical cell, using a platinum (Pt) wire as the counter electrode, a saturated calomel electrode (SCE) as the reference electrode and the sample as working electrode. Measurements have been performed on MWCNT films grown on a Si(111) substrate and a cut piece of CNT-sponge. A 0.5 M KI and 0.01M I_2_ in acetonitrile solution was used as the electrolyte. Samples were irradiated with light coming from a 200 W Xe lamp (Osram) equipped with a monochromator (λ > 300 nm) and a PG-310 potentiostat (HEKA Elektronik, Lambrecht, Germany) measured the photo-current. The intensity of the photo-current signal was normalized to the irradiated sample area and the incident light power and expressed as IPCE (incident-photon-to-current efficiency) [27–29]. The light intensity near the electrode surface was estimated to be about 50 mW·cm^−2^ by azobenzene actinometry [30].
